# Escin Chemosensitizes Human Pancreatic Cancer Cells and Inhibits the Nuclear Factor-kappaB Signaling Pathway

**DOI:** 10.1155/2013/251752

**Published:** 2013-10-27

**Authors:** A. Rimmon, A. Vexler, L. Berkovich, G. Earon, I. Ron, S. Lev-Ari

**Affiliations:** ^1^Laboratory of Herbal Medicine and Cancer Research, Institute of Oncology, Tel Aviv Sourasky Medical Center, 6 Weizmann Street, 64239 Tel Aviv, Israel; ^2^Sackler Faculty of Medicine, Tel Aviv University, Tel Aviv, Israel

## Abstract

*Background*. There is an urgent need to develop new treatment strategies and drugs for pancreatic cancer that is highly resistant to radio-chemotherapy. *Aesculus hippocastanum* (the horse chestnut) known in Chinese medicine as a plant with anti-inflammatory, antiedema, antianalgesic, and antipyretic activities. The main active compound of this plant is Escin (C_54_H_84_O_23_). *Objective*. To evaluate the effect of Escin alone and combined with chemotherapy on pancreatic cancer cell survival and to unravel mechanism(s) of Escin anticancer activity. *Methods*. Cell survival was measured by XTT colorimetric assay. Synergistic effect of combined therapy was determined by CalcuSyn software. Cell cycle and induction of apoptosis were evaluated by FACS analysis. Expression of NF-**κ**B-related proteins (p65, I**κ**B*α*, and p-I**κ**B*α*) and cyclin D was evaluated by western blot analysis. *Results*. Escin decreased the survival of pancreatic cancer cells with IC_50_ = 10–20 M. Escin combined with gemcitabine showed only additive effect, while its combination with cisplatin resulted in a significant synergistic cytotoxic effect in Panc-1 cells. High concentrations of Escin induced apoptosis and decreased NF-**κ**B-related proteins and cyclin D expression. *Conclusions*. Escin decreased pancreatic cancer cell survival, induced apoptosis, and downregulated NF-**κ**B signaling pathway. Moreover, Escin sensitized pancreatic cancer cells to chemotherapy. Further translational research is required.

## 1. Introduction

Pancreatic cancer is considered to be one of the most aggressive forms of human cancers. It is the fourth leading cause of cancer deaths in the United States, its median survival rate is 4–6 months, and the overall 5-year survival rate is only 6% [1, 2]. Moreover, it is highly resistant to apoptosis-inducing therapy, such as radio- and chemotherapy [3, 4]. Thus, there is an urgent need to develop new treatment strategies in order to reduce the high mortality rates of these patients. According to ethnobotany pharmacopeia, active compounds of many plants are used for medical needs and provide additional therapeutic effects to modern medicine. One meta-analysis of drugs that were developed between 1981 and 2001 revealed that natural products constitute a significant percentage of those drugs [5]. Many antitumor agents that are now in clinical oncology practice are of natural origin, such as taxanes (docetaxel, paclitaxel), Vinca alkaloids (vindesine, vinblastine, vincristine), and anthracyclines (idarubicin, daunorubicin, epirubicin), indicating a promising future in the use of natural products from plants as anti-tumor agents [6].

Natural compounds, such as curcumin [7–9] and moringa [10], or polybotanical compounds, such as BreastDefend and ProstaCaid [11, 12] were found to potentiate the effect of standard chemotherapy. One of such natural products is the extract of *Aesculus hippocastanum* (the horse chestnut) seeds that has been used in China as an analgesic and an antipyretic agent [13]. The main active compound of this extract is Escin (C_54_H_84_O_23_), a penta cyclic triterpene. It is as a saponin mixture that exists in two forms, *α* and *β*. The *β* form was found to be the active one, exhibiting anti-inflammatory [14] and antiedema activities [15]. Recent studies found it to have an effect on induction of apoptosis in different cancer cell lines [16–18]. As a natural product, Escin is considered to be relatively safe to consume as well as being affordable. It is currently under clinical trials in patients with HIV-1 [19], for the treatment of blunt impact injuries [20] and cutaneous pruritus [21]. The mechanism of its anti-inflammatory effects is not fully understood [22], but it might be connected to an inhibition of HIV-1 protease [23], suppression of adhesion molecules on endothelial cells [24], and prevention of hypoxia-induced adhesiveness of neutrophils to endothelial cells [25].

 The nuclear factor-kappaB (NF-**κ**B) signaling pathway is known to be constitutively activated in chronic inflammation that increases cancer risk and promotes cancer progression [26]. This factor is overexpressed in a large number of epithelial and hematologic malignancies. It is constitutively active in pancreatic cancer [27] and is linked with cell proliferation, invasion, angiogenesis, metastasis, and suppression of apoptosis [28], as well as with chemoresistance [29]. Therefore, NF-*κ*B is strongly considered as being a potential molecular target for cancer therapy [30]. 

Harikumar et al. [31] found that Escin chemosensitizes human tumor (leukemia and multiple myeloma cells) through the inhibition of nuclear factor-kappaB signaling pathway. We therefore examined the influence of Escin on the NF-**κ**B signaling pathway in pancreatic cancer cells. It is possible that Escin's activity might be linked to the NF-**κ**B signaling pathway. Regulating this pathway might lead to the induction of apoptosis and the inhibition of survival. As is seen in some natural products, Escin may also potentiate the effect of standard chemotherapy, such as cisplatin and gemcitabine which are widely used for the treatment of pancreatic cancer. The specific aims of this study were to assess the effect of Escin alone and in combination with chemotherapy on pancreatic cancer cell survival and to examine whether this effect is associated with downregulation of the NF-*κ*B signaling pathway.

## 2. Materials and Methods

### 2.1. Cell Lines

The human pancreatic cancer cell lines Panc-1, COLO 357, and MIA-Paca were obtained from the American Type Culture Collection. “A true long-term cancer cell line, p34,” which that had been developed from a pleural effusion from a pancreatic cancer patient was also used. The cells were maintained in Dulbecco's modified Eagle's medium (DMEM) supplemented with 10% fetal bovine serum (FBS), antibiotics, and pyruvic acid at 37°C in a 5% CO_2_ humidified atmosphere.

### 2.2. Reagents

The Escin tested in this study as anticancer treatment alone or in combination with chemotherapy was received as a powder from Sigma-Aldrich (St. Louis, MO, USA). For stock solution (100 mM), Escin was diluted in dimethyl sulfoxide (DMSO, Merck, Israel) and stored as small aliquots at −20°C. Before each experiment, Escin was diluted in cell culture medium as required. DMEM, FBS, penicillin, streptomycin, and pyruvic acid were obtained from Biological Industries (Beit HaEmek, Israel). Antibodies against p65, I*κ*B*α*, and cyclin D were obtained from Santa Cruz Biotechnology (Santa Cruz, CA, US). Phospho-I*κ*B*α* (p-I*κ*B*α*) antibody was obtained from Cell Signaling Technology (USA). An antibody against actin was obtained from MP Biomedicals (USA).

### 2.3. Cell Survival Assay

For the evaluation of cell survival following the tested treatments, the cells (1.5 × 10^3^ cells per well, 200 *μ*L) were plated in 96-well plates and allowed to attach overnight. The 24 hr cell cultures were used in all the experiments. The medium was replaced with medium with or without selected concentrations of Escin in triplicates and incubated for an additional 72 hrs. The number of viable cells was evaluated using XTT-based colorimetric cell proliferation assay (Biological Industries). After 3-4 hours of cell incubation with a medium containing the XTT reagent, the optical density was measured at 450 nm by a TECAN microplate reader (Sunrise, Switzerland). The rate of cell survival following treatment was expressed as a percentage of viable cells relative to control values.

### 2.4. Analysis of the Synergistic Effect of Treatment

 In order to determine the synergistic effect, the data were analyzed with CalcuSyn software which is based on the Chou and Talalay's equations [32] as follows.

The combination index (CI) was calculated using the following equation:

(1) $$\begin{array}{c} \text{CI}=\frac{{\left(D\right)}_{1}}{{\left(D_{x}\right)}_{1}}+\frac{{\left(D\right)}_{2}}{{\left(D_{x}\right)}_{2}}, \end{array}$$

where (*D*)_1_ and (*D*)_2_ are the doses of drug 1 and drug 2 in a mixture that inhibits the system *x* percent, and (*D*_*x*_)_1_ and (*D*_*x*_)_2_ are the doses of the drugs that were given individually that inhibited the system *x* percent. According to the software, CI < 1, = 1, and >1 indicates synergism, additive effect, and drugs antagonism, respectively.

The median-effect equation *f*_*a*_/*f*_*u*_ = (*D*/*D*_*m*_)_*m*_ was used in order to calculate (*D*_*x*_)_1_ and (*D*_*x*_)_2_, where *f*_*a*_ is the fraction affected by the dose, *f*_*u*_ is the fraction unaffected by the dose, and *f*_*u*_ = 1 − *f*_*a*_. *D* is the dose of drug, and *D*_*m*_ is the median-effect dose signifying the potency. *D*_*m*_ was determined from the *x*-intercept of the median-effect plot, and *m* is an exponent determined by the slope of the median-effect plot.

### 2.5. Cell Cycle Analysis

The distribution of Panc-1 cells in the cell cycle was evaluated by flow cytometry. The cells (2 × 10^6^) were treated with Escin for 24 hours, after which they were collected, washed with cold phosphate-buffered solution (PBS), and fixed in ice-cold 70% ethanol. The samples were kept at −20°C for 24–48 hours. Before the analysis, the cells were washed with cold PBS and then suspended in PBS solution containing 0.1% Triton-X and 30 mg/mL DNAse-free RNAse A (Sigma) for 6 hours at room temperature. Several minutes before the cells were analyzed, propidium iodide (Sigma) in PBS was added at a final concentration of 10 *μ*g/mL. The samples were then analyzed by flow cytometry using an FACSCallibur instrument (BD Bioscience, San Jose, CA). Data were processed with BD Bioscience software. Data for at least 10,000 cells were collected for each data file. Necrotic cells that had been detected by counting cells following staining with trypan blue before fixation were excluded from the calculation of apoptotic cells. All experiments were repeated 2-3 times, and the results were calculated as mean ± SE.

### 2.6. Immunoblotting Analysis

The cells plated in 10 cm dishes were allowed to attach and proliferate for up to 48 hours at 37°C in a 5% CO_2_ humidified atmosphere. The culture medium was then replaced with fresh medium supplemented with Escin (10–30 *μ*M) for 2 hours. Whole-cell protein samples for immunoblotting were prepared using the proteoJET mammalian cell lysis reagent according to standard protocol (Fermentas Life Sciences, Abu-gosh, Israel). Cytoplasmic and nuclear extracts for immunoblotting were prepared using the NucBuster protein extraction kit (Novagen). The protein concentration was determined using the Bradford assay. Each sample (100 *μ*g) was subjected to electrophoresis in 10% SDS-PAGE and then transferred to pure nitrocellulose blotting membranes (Millipore, Bedford, MA). Membranes were blocked for 1 hour at room temperature with Tris (hydroxymethyl) amino methane saline (TBS) containing 0.05% Tween 20 and 5% nonfat skim milk powder (BD Bioscience). The membranes were then incubated at room temperature in PBS containing 5% milk powder and the corresponding antibodies (1 : 1000 dilutions) against p65, I*κ*B*α*, p-I*κ*B*α*, cyclin D, and actin. The membranes were washed in TBS 0.05% Tween 20 and incubated with either goat anti-rabbit or goat anti-mouse (1 : 2000) secondary antibodies conjugated to horseradish peroxidase (Santa Cruz Biotechnology, Santa Cruz, CA). Detection of expression of proteins was done by using SuperSignal West Pico reagent from Pierce BioLynx Inc. (Brockville, Ontario, Canada). 

### 2.7. Statistical Analysis

The results for each variant in the *in vitro* experiments were represented as an average from 2–4 experiments, and each arm was typically performed in triplicate. The mean values and standard errors were calculated for each point from the pooled normalized data. The difference between the arms was analyzed using the two-tailed Student *t*-test and considered as being statistically significant if *P* < 0.05 (marked in the figures as ∗) and especially *P* < 0.01 (marked in the figures as ∗∗).

## 3. Results

### 3.1. Effect of Escin on Survival of Pancreatic Cancer Cells

**Figure 1:**
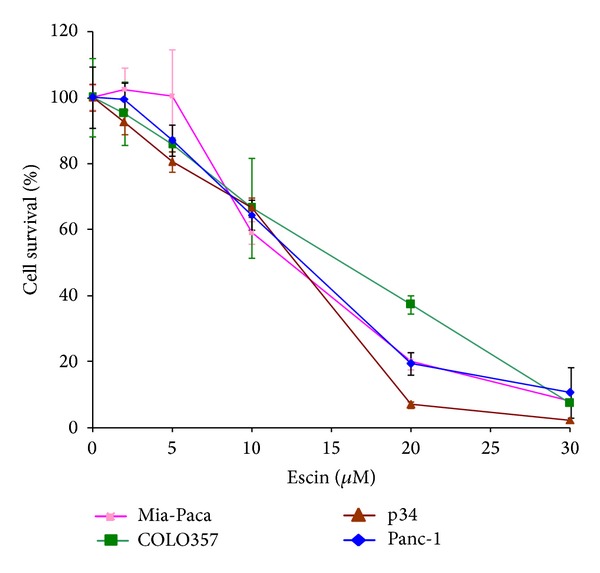
Effect of Escin on survival of cultured pancreatic cancer cells. Panc-1, MIA-Paca, COLO 357, and p34 cells were plated in 96-well plates and treated with Escin (2–30 *μ*M) for 3 days. Cell survival was assessed by XTT assay in 3 independent experiments conducted in triplicates.

The effect of Escin on the survival of cultured pancreatic carcinoma cells was evaluated by using four cancer cell lines, Panc-1, p34, MIA-Paca, and COLO 357. The cells were treated with 2–30 *μ*M of Escin for 72 hours. The treatment with 10 *μ*M Escin decreased the survival of Panc-1, COLO 357, and p34 cell lines to 65%, while the survival of Mia-Paca was less than 60% (Figure 1). The treatment of COLO 357, cells with 20 *μ*M of Escin decreased the cell survival to 40%. This concentration of Escin decreased the survival of Panc-1 and Mia-Paca cells even more significantly (to 20%). The p34 cells were more sensitive to this treatment, showing a more than 90% effect. An increase of Escin concentration up to 30 *μ*M resulted in the almost total decrease of cell survival (>80%) for all cell lines tested. These findings demonstrated that Escin is a potent inhibitor of the growth of the pancreatic cancer cells.

### 3.2. Effect of Escin on the Induction of Apoptosis in Panc-1 Cells

**Figure 2:**
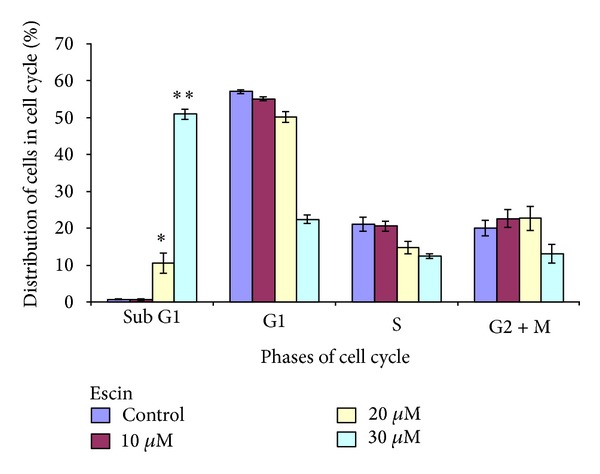
Effect of Escin on the distribution of Panc-1 cells in the cell cycle. The samples of the cells treated for 24 hours with various Escin concentrations were prepared and stained by propidium iodide immediately before flow cytometry analysis. The difference between the nontreated and treated cells in the induction of apoptosis was significant. *P < 0.05 and **P < 0.01.

Since Escin treatment resulted in a significant decrease of cell survival, we examined its effect on apoptosis by measuring the percentage of cells with subdiploid DNA content, which is characteristic of apoptosis, by using flow cytometry (FACS) analysis. Treatment of Panc-1 cells with 10 *μ*M of Escin had no effect on the distribution of the cells in the cell cycle (Figure 2). The treatment with higher concentrations of Escin, however, resulted in a significant increase of the fraction of apoptotic cells with a sub-G1 content of DNA that reached 10% at 20 *μ*M (*P* < 0.05) and 50% at 30 *μ*M (*P* < 0.01).

### 3.3. Effect of Escin on the Expression of NF-**κ**B-Related Proteins

**Figure 3:**
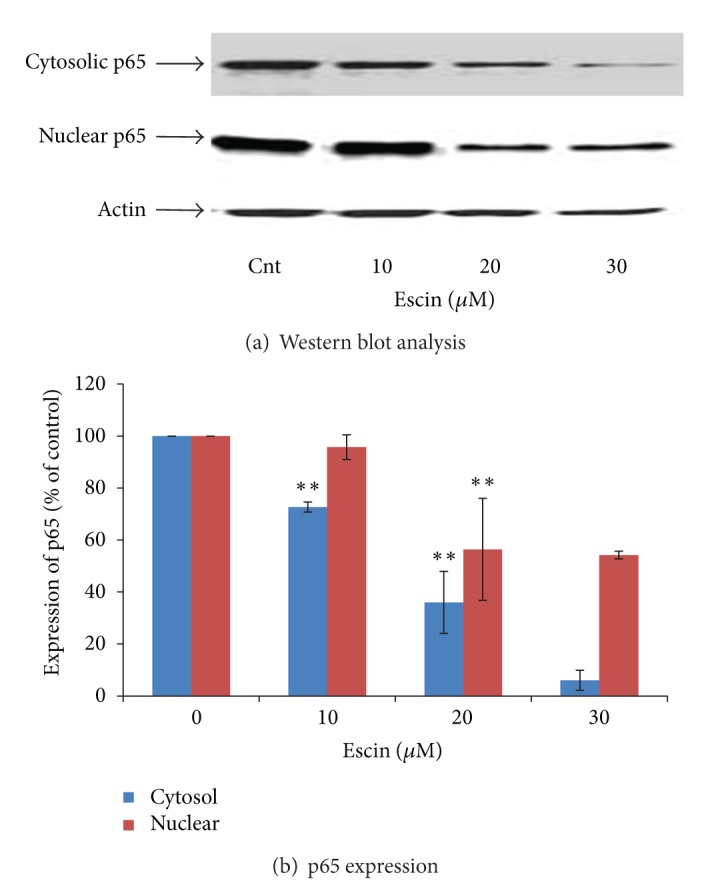
Effect of Escin on the expression of p65 in the cytosol and nucleus of Panc-1 cells. (a) Western blot analysis. (b) Densitometry results averaged from 3 independent experiments. **P < 0.01 versus control.

**Figure 4:**
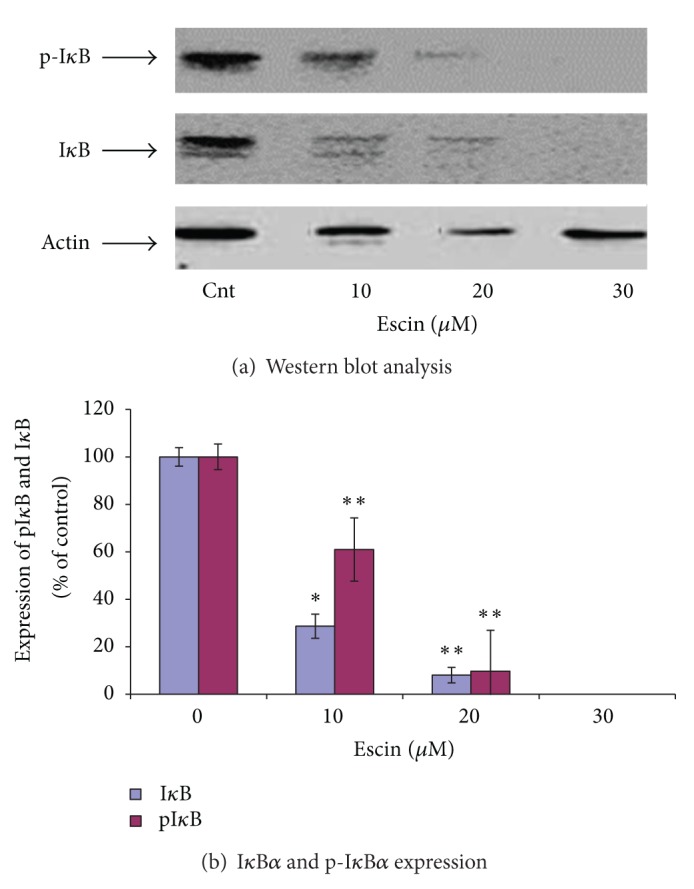
Effect of Escin on the expression of p-I*κ*B*α* and I*κ*B*α* in Panc-1 cells. (a) Western blot analysis. (b) Densitometry results averaged from 3 independent experiments. ^#^The expressions of p-I*κ*B*α* and I*κ*B*α* were not detectable with Escin 30 *μ*M.

We next investigated whether the inhibitory effect of Escin on pancreatic cancer cell survival is mediated through the alteration of NF-**κ**B-related proteins. Protein extracts of nontreated and Escin-treated Panc-1 cells were analyzed by Western blot assay to assess their phospho-I**κ**B and p65 expressions. Escin significantly reduced the level of p65 in both the cytosol and in the nucleus (Figures 3(a) and 3(b)), as well as the total level of I*κ*B*α* and phospho-I*κ*B*α*, both in a dose-dependent manner (Figures 4(a) and 4(b)). 

**Figure 5:**
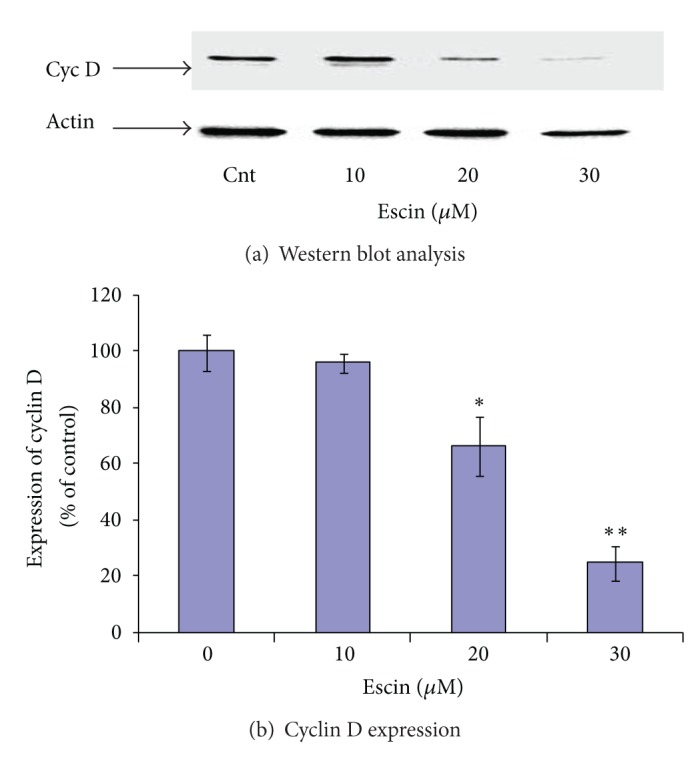
Effect of Escin on the expression of cyclin D in Panc-1 cells. (a) Western blot analysis. (b) Densitometry results averaged from 3 independent experiments.

We also examined the effect of Escin on the level of cyclin D that is associated with cell progression and proliferation. As shown in Figures 5(a) and 5(b), Escin significantly decreased the level of cyclin D in a dose-dependent manner.

### 3.4. Combined Effect of Escin and Chemotherapy Agents on Panc-1 Cell Survival

**Figure 6:**
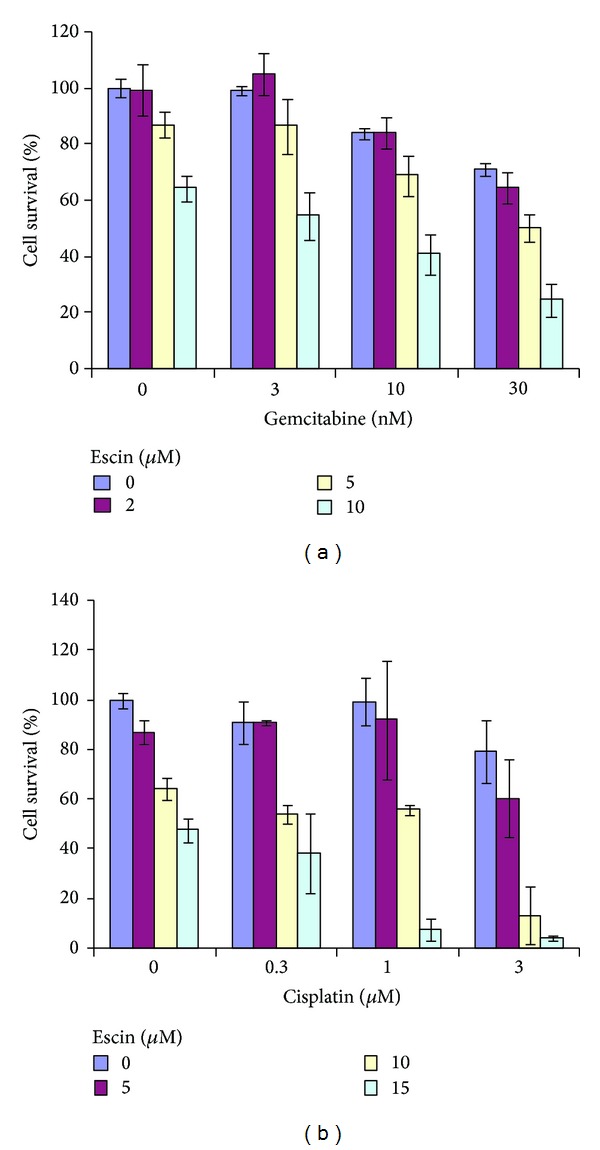
Effect of Escin combined with gemcitabine or cisplatin on survival of Panc-1 cells. Panc-1 cells were plated in 96-well plates and treated with gemcitabine (3, 10, and 30 nM) for 3 days. Cell survival was assessed using the XTT-based cell proliferation assay. Three independent experiments were conducted in triplicate.

**Figure 7:**
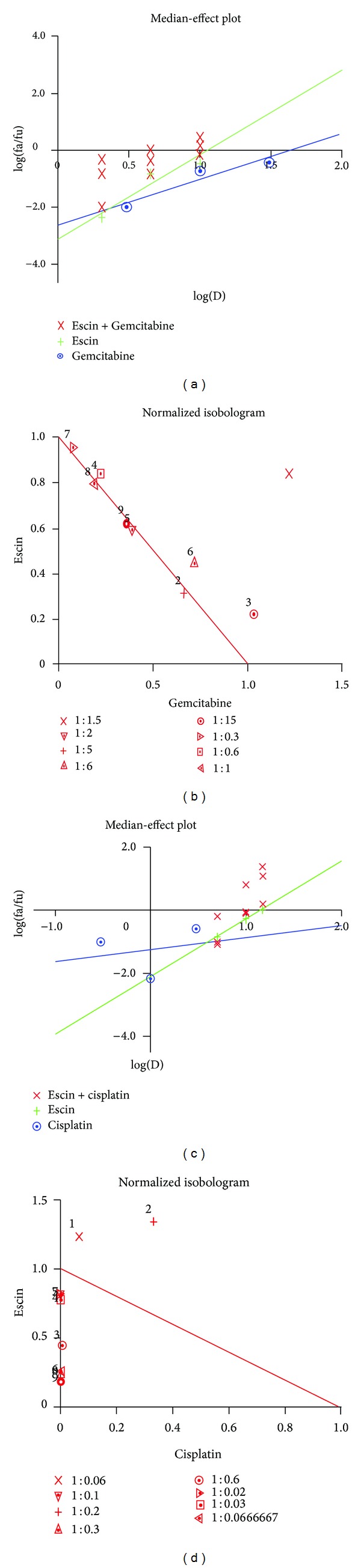
Analysis of combined effect of chemotherapy and Escin. Escin + gemcitabine, median-effect plot (a); Escin + gemcitabine, normalized isobologram (b); Escin + cisplatin, median plot effect (c), Escin + cisplatin, normalized isobologram (d).

Having found pancreatic cancer cells to be sensitive to Escin, we further examined the combined effect of Escin with two chemotherapeutic drugs currently used in clinical oncology, cisplatin and gemcitabine. The doses were chosen in the range of low inhibitory effect of the drugs alone: specifically, Escin at 2, 5, 10, and 15 *μ*M, gemcitabine at 3, 10, and 30 nM, and cisplatin at 0.3, 1, and 3 *μ*M. Panc-1 cells were treated for 72 hours with selected combinations of those agents. The combined effect of Escin with gemcitabine on the survival of Panc-1 cells was higher than the cytotoxic effect of gemcitabine or Escin alone at most of the combinations tested (Figure 6(a)). There was a significant inhibitory effect of Escin combined with cisplatin at all tested combinations, and it was much more pronounced compared to the cytotoxic effect of each agent alone (Figure 6(b)). In order to evaluate the mode of cooperation of the agents tested, the data of combined treatments were analyzed using CalcuSyn software that allows us to calculate the combination index (CI) for each combination of the drugs tested and to construct isobolograms. According to the software, a combined treatment is additive when CI > 1, additive if CI = 1, and synergistic when CI < 1. CalcuSyn applied the experimental data to calculate median effect plot curves for each treatment and isobolograms for the combined treatments (Figures 7(a) and 7(b)).

As shown in Table 1, the treatments of the Panc-1 cells by Escin in combination with gemcitabine resulted mainly in an additive effect (in the range of CI ~ 1). Contrarily, the treatment of the Panc-1 cells with Escin in combination with cisplatin resulted mainly in low CI < 1 values that are characteristic of a synergistic effect. Notably, the synergistic effect was significantly dependent on the doses of both Escin and cisplatin. While cisplatin 0.3 *μ*M alone did not inhibit cell survival, the effect was weakly synergistic and increased when combined with Escin dose. There was a more significant dose-dependent synergistic effect when higher concentrations of Escin were combined with 1 *μ*M cisplatin: the CI was <0.5 at all tested Escin doses.

## 4. Discussion

The results of this study show that Escin significantly decreased the growth of human pancreatic carcinoma cells, inhibited NF-**κ**B signaling pathways, and sensitized pancreatic cancer cells to the cytotoxic effect of chemotherapy. Several medicinal herbs with anti-inflammatory properties were found to have a role in the prevention and treatment of cancer. Therefore, we hypothesized that Escin, which is derived from horse chestnut seeds and which had already exhibited anti-inflammatory activities [14] could be used as an effective treatment against pancreatic cancer cells, either when administered alone or in combination with existing treatment protocols. Our *in vitro* experiments revealed that Escin significantly inhibited the growth of all of the pancreatic cancer cells tested (Panc-1, Mia-Paca, COLO357, and P34) in a dose-dependent manner. 

One of the reasons for the inhibiting effect of Escin on cancer cell proliferation may be an induction of apoptosis that had also been observed after other anticancer treatments. We used FACS analysis to measure the proportion of the intact and treated cells characterized by the sub-G1 content of DNA, hallmark of apoptosis. FACS analysis of Escin-treated Panc-1 cells revealed that the fraction of apoptotic cells reached 10% at the 20 *μ*M dose and that about 50% of the cells were apoptotic at the 30 *μ*M dose (Figure 2). Similar data on the induction of apoptosis by Escin were shown by Zhou et al. [33] and are in good correlation with similar activity of other natural products. For example, we had shown that curcumin decreased cell survival and enhanced the induction of apoptosis in lung and pancreatic adenocarcinoma cells [33]. The increased induction of apoptosis may partly explain Escin's inhibitory effect on cell survival.

Another reason for the resultant cell death may be damage to the cellular membrane. The trypan blue staining of intact cells demonstrated 2-3% of stained cells, among this population, while an increase of the proportion of these cells of up to 10% was observed following Escin 20 *μ*M treatment (data not shown). 

It is well known that the nuclear factor-kappaB (NF-**κ**B) signaling pathway plays a key role both in inflammation and in cancer development and progression, including cancer cell proliferation, invasion, angiogenesis, and metastasis. Given the evidence that Escin contains anti-inflammatory properties [14], we assessed the effect of Escin on NF-**κ**B proteins, such as p65, I*κ*B*α*, and phospho-I*κ*B*α*. Our findings on the high expression of these proteins in Panc-1 cells (Figures 3–5) are in good correlation with those reported by others [34]. Escin treatment decreased the cytosolic and nuclear levels of p65 (Figure 3), as well as total I*κ*B*α* and phospho-I*κ*B*α* levels in a dose-dependent manner (Figure 4). We presume that the reduction of p65 and I*κ*B*α* by Escin may be the result of increased proteosome degradation of these proteins that was demonstrated following tylophorine analogue treatment of several pancreatic cancer cell lines [35]. The reduction of I*κ*B*α*, a substrate of phospho-I*κ*B*α*, led to the reduction of phospho-I*κ*B*α* levels. Our data of a downregulation of the NF-**κ**B signal transduction pathway is similar to the finding of Wang et al. on pancreatic cancer cells [36]. Furthermore, other studies [37, 38] demonstrated that the inhibition of cancer cells proliferation and invasion by natural products is correlated with downregulation of NF-**κ**B proteins, such as nuclear and cytoplasmic p65. As such, our findings on NF-**κ**B downregulation may explain the decrease in pancreatic cancer cell survival following Escin treatment and the increase of the proportion of apoptotic cells observed in the current experiments.

An additional explanation for Escin's inhibitory effect on the survival of pancreatic cancer cells may be the down-regulation of the level of cyclin D that is known to be responsible for cell cycle progression and cell proliferation [39–41]. This is supported by the finding of a significant diminishing of the cyclin D level in Panc-1 cells following Escin treatment (Figure 5).

In order to avoid chemoresistance of tumors, most modern protocols of cancer treatment include the combination of several cytotoxic drugs with different mechanism(s) of anticancer effects [42]. Several reports correlated the chemo-resistance of pancreatic cancer with high activity of the NF-**κ**B signal transduction pathway [43–45]. For example, Harikumar et al. [46] showed that suppression of NF-**κ**B pathway by sesamin resulted in potentiation of apoptosis induced by TNF-*α*.

We therefore tested the efficacy of Escin in combination with chemotherapeutic drugs which are widely used in oncology practice, cisplatin, and gemcitabine [47, 48]. The efficacy of these drugs in combination with Escin had been tested on Panc-1 cells that are known to be moderately resistant to chemotherapeutic drugs [49].

As expected, both cisplatin and gemcitabine reduced the survival of Panc-1 cells in a dose-dependent manner (data not shown). We now demonstrated, for the first time, that the combined effect of Escin with these drugs was more effective than the use of each agent alone (Figure 6). We analyzed the results with CalcuSyn software in order to understand the mode of the interaction of the tested drugs and revealed that most of the combinations of Escin with cisplatin have a synergistic effect on pancreatic cell survival (Figure 7). The greatest synergistic effect (CI = 0.256 and 0.186) was found when the highest concentrations of Escin (10 and 15 *μ*M) were combined with the highest concentration of cisplatin (3 *μ*M), while the synergistic effect was lower (CI = 0.454) or almost additive (CI = ~0.8) at other combinations. The combined effect was additive for the combinations of the smallest concentration of Escin (5 *μ*M) with smaller concentrations of cisplatin. The same analysis of Escin treatment combined with gemcitabine demonstrated an only additive effect (CI = ~1.0) on cell survival (Figure 7). The difference in activity of the tested drug combinations may be explained by the differences in the mechanisms of anticancer activity of the chemotherapeutic drugs used. Cisplatin is an alkylating agent that induces cell death by causing cross-linking in DNA, whereas gemcitabine is a pyrimidine antimetabolite that contributes to the inhibition of DNA synthesis. The higher efficacy of Escin and cisplatin combination that was observed here may be explained in part by the high increase in the proportion of sub-G1 cells following Escin treatment (Figure 2) and the known DNA damage caused by cisplatin.

Finally, the chemosensitization of pancreatic tumor cells appears to be mediated through the ability of Escin to modify cell-signaling molecules, including cell proliferating proteins, such as cyclin D and members of the NF-*κ*B signaling pathways, as described for resveratrol by Gupta et al. [50] whose *in vitro* studies found that resveratrol inhibited the proliferation of human PaCa pancreatic cell lines, synergized the apoptotic effects of gemcitabine, and inhibited both the constitutive activation of NF-*κ*B and the expression of Bcl-2, Bcl-xL, COX-2, cyclin D1, MMP-9, and VEGF.

## 5. Conclusion

We found that treatment with Escin decreased pancreatic cancer cell survival, induced apoptosis, reduced the NF-**κ**B signal transduction pathway, and resulted in the sensitization of pancreatic cancer cells to chemotherapeutics agents. Further translational research of Escin is required to assess its role as an adjuvant therapy agent in the clinical setting.

## Figures and Tables

**Table 1:** Analysis of a mode of combined treatment with Escin + gemcitabine or Escin + cisplatin.

Escin (*µ*M)	Gemcitabine (nM)	Combination index (CI)	Escin (*µ*M)	Cisplatin (*µ*M)	Combination index (CI)
2	3	2.07	5	0.3	1.30
5	3	1.06	10	0.3	0.78
10	3	1.04	15	0.3	0.82
2	10	0.97	5	1	1.68
5	10	0.98	10	1	0.81
10	10	0.98	15	1	0.27
2	30	1.25	5	3	0.45
5	30	1.17	10	3	0.26
10	30	0.98	15	3	0.19
